# The Effects of *Eucheuma cottonii* on Signaling Pathway Inducing Mucin Synthesis in Rat Lungs Chronically Exposed to Particulate Matter 10 (PM_10_) Coal Dust

**DOI:** 10.1155/2013/528146

**Published:** 2013-10-08

**Authors:** Nia Kania, Elly Mayangsari, Bambang Setiawan, Dian Nugrahenny, Frans Tony, Endang Sri Wahyuni, M. Aris Widodo

**Affiliations:** ^1^Department of Pathology, Ulin General Hospital, Faculty of Medicine, University of Lambung Mangkurat, Banjarmasin,South Kalimantan, Indonesia; ^2^Laboratory of Pharmacology, Faculty of Medicine, Brawijaya University, Malang, East Java, Indonesia; ^3^Department of Medical Chemistry and Biochemistry, Faculty of Medicine, University of Lambung Mangkurat, Banjarmasin, South Kalimantan, Indonesia; ^4^Department of Marine, Faculty of Fisheries, University of Lambung Mangkurat, Banjarbaru, South Kalimantan, Indonesia; ^5^Laboratory of Physiology, Faculty of Medicine, Brawijaya University, Malang, East Java, Indonesia

## Abstract

This study was aimed at investigating the effects of *Eucheuma cottonii* (EC) in oxidative stress and the signaling for mucin synthesis in rat lungs chronically exposed to coal dust. Coal dust with concomitant oral administration of ethanolic extract of EC at doses of 150 (EC_150_) or 300 mg/kg BW (EC_300_) compared to exposed to PM_10_ coal dust at doses of 6.25 (CD_6.25_), 12.5 (CD_12.5_), or 25 mg/m^3^ (CD_25_) (an hour daily for 6 months) and nonexposure group (control). The malondialdehyde (MDA), epidermal growth factor (EGF), transforming growth factor (TGF)-**α**, epidermal growth factor receptor (EGFR), and MUC5AC levels were determined in the lung. The administration of EC_300_ significantly (*p* < 0.05) reduced the MDA levels in groups exposed to all doses of coal dust compared to the respective coal dust-exposed nonsupplemented groups. Although not statistically significant,EC reduced the EGF levels and EGFR expressions in CD_12.5_ and CD_25_ groups and decreased the TGF-**α**, level and MUC5AC expression in CD_25_ group compared to the respective coal dust-exposed nonsupplemented groups. EC was able to decrease oxidative stress and was also able to decrease signaling for mucin synthesis, at least a part, via reducing the ligand in chronic coal dust exposure.

## 1. Introduction

In healthy individuals, inhaled foreign materials become entrapped in the mucus and are cleared by mucociliary transport and by coughing. However, in many chronic inflammatory airway diseases, excessive mucus is produced and is inadequately cleared, leading to mucous obstruction and infection [1].

The inhalation of occupational and atmospheric coal dust has been reported to significantly contribute to the development of several respiratory disorders, including infection, inflammation, and remodelling of the lungs [2]. Several studies have found that coal dust is radical itself, and it also produces free radicals [3], thus increasing oxidative stress in rats lung [4, 5] and human blood [6]. Expression of MUC5AC, a major secreted, gel-forming respiratory tract mucin, is closely linked to goblet cell metaplasia and mucus hypersecretion [7]. Oxidative stress may regulate gene expression at both transcriptional and posttranscriptional levels. Oxidative stress regulates MUC5AC mRNA expression via activation of the EGFR [8, 9] and by an alternative mechanism, post-transcriptional regulation [10].

In recent years, marine resources have attracted attention as a source of bioactive compounds for the development of new drugs and healthy foods [11]. In particular, seaweeds are a very important and commercially valuable resource for the food industry and are used in traditional medicine [12]. The abundantly cultivated edible red seaweed, *Eucheuma cottonii* (*Kappaphycus alvarezi*), grows very rapidly in pristine water in Southeast Asia and can be harvested every 45 days for human use. It contains high amounts of dietary fibers, minerals, vitamins, antioxidants, polyphenols, phytochemicals, proteins, and polyunsaturated fatty acids and has medicinal uses [13]. *E. cottonii* is one of the main seaweeds species cultivated in Tamiang Gulf of South Kalimantan. Previous studies showed that *E. cottonii* has the best antihyperlipidemic and *in vivo* antioxidant activity, which significantly reduced body weight gain, elevated erythrocyte GSH-Px, and reduced plasma lipid peroxidation of high fat diet rats towards the values of normal rats [14]. The polyphenol-rich *E. cottonii* has tumor-suppressive activity via apoptosis induction, downregulating the endogenous estrogen biosynthesis, and improving antioxidative status in the rats [15].

In this study, we investigated the changes in oxidative stress, the levels of EGF and TGF-*α*, and the expressions of EGFR and MUC5AC in rat lungs chronically exposed to PM_10_ coal dust. We hypothesized that such exposure changes the EGFR ligand and its downstream signaling, and the administration of *E. cottonii* can significantly reduce such effects.

## 2. Materials and Methods

### 2.1. Preparation and Extraction of *E. cottonii*

*E. cottonii* was harvested from the coastal areas of Tamiang, Kotabaru (South Kalimantan, Indonesia). X-ray Fluorescence analysis of this species found no toxic minerals (data not shown). The preparation and extraction of the seaweed were performed according to the method of Fard et al. [16]. The fresh seaweed was thoroughly washed with distilled water, and their holdfasts and epiphytes were removed. The cleaned seaweed was then dried at 40°C in dark room for 3 days and grounded into fine powder using a miller. The powder was stored at −20°C in airtight containers wrapped by aluminum foil. Then, the powder (200 g) was mechanically stirred with 1000 mL of 80% (v/v) ethanol at room temperature (RT) for 24 h and filtered. The residue was then dissolved in 3000 mL of distilled water and stirred at RT for 8 h. Subsequently, the extract was then filtered and concentrated under negative pressure at 40 and 70°C for 1 h, respectively. The extract was oven dried at 40°C overnight to produce powdered extracts and then stored at −20°C in airtight containers until application.

### 2.2. Determination of Antioxidant Activity (Scavenging Activity of DPPH Radical)

The antioxidant activity was evaluated by diphenylpicrylhydrazyl (DPPH) free radical scavenging assay. DPPH is a molecule containing a stable free radical. In the presence of an antioxidant, which can donate an electron to DPPH, the purple color typical for DPPH radical decays, and the change in absorbance is then read at 517 nm using the spectrophotometer. The assay was performed according to the method described by Brand-Williams et al. [17]. Various concentrations (6.25, 12.5, 25, 50, and 100 *μ*g/mL) of EC were prepared and similar concentrations of catechin were used as a positive control. The assay mixture contained 500 *μ*L of the sample extract, 125 *μ*L of prepared DPPH (1 mM in ethanol), and 375 *μ*L of solvent (ethanol). After 30 min incubation at 25°C, the absorbance was measured at 517 nm. The radical scavenging activity was then calculated from the following equation: Radical  scavenging  activity  (%) = [(Abs_control_ − Abs_sample_)/Abs_control_] × 100, where Abs_control_ is the absorbance of DPPH radical + solvent; Abs_sample_ is the absorbance of DPPH radical + sample extract/catechin [18, 19].

### 2.3. Animals

Eighty male Wistar albino rats, 16 weeks of age, weighing 170–200 gram, were used for this study. Animals were housed in a clean wire cage and maintained under standard laboratory conditions with temperature of 25 ± 2°C and dark/light cycle 12/12 h. Standard diet and water were provided ad libitum. Animals were acclimatized to laboratory conditions for one week prior to the experiment. Animal care and experimental procedures were approved by the institutional ethics committee of Faculty of Medicine, Brawijaya University, Malang, Indonesia.

### 2.4. Coal Dust Preparation

Coal dust preparation was performed as described in our previous study [20]. Two kilograms of subbituminous gross coals obtained from coal mining area in South Kalimantan, Indonesia, were pulverized by Ball Mill, Ring Mill, and Raymond Mill in Carsurin Coal Laboratory of Banjarmasin. Coal dust particles were then filtered by Mesh MicroSieve (BioDesign, New York, NY, USA) to obtain particles with diameter less than 10 *μ*m (PM_10_). Subsequently, PM_10_ coal dust was characterized by scanning electron microscope (SEM), X-ray fluorescence, and X-ray diffraction in the Physic and Central Laboratory, Faculty of Mathematics and Natural Science, University of Malang, Indonesia.

### 2.5. Coal Dust Exposure

**Figure 1:**
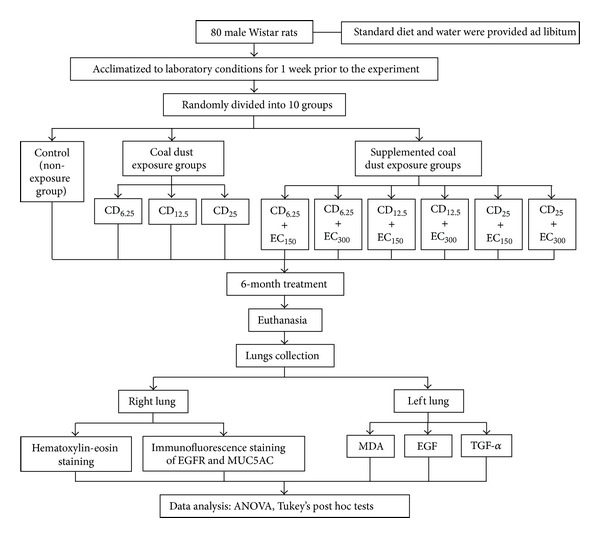
The schematic design of this study. Eighty male Wistar rats were randomly divided into ten groups. One group is a non-exposure group (control). Three groups were exposed to PM_10_ coal dust at doses of 6.25 (CD_6.25_), 12.5 (CD_12.5_), or 25 mg/m^3^ (CD_25_) an hour daily for 6 months. Six groups were exposed to coal dust with concomitant oral administration of *Eucheuma cottonii* at doses of 150 (EC_150_) or 300 mg/kg BW (EC_300_).

Eighty male Wistar rats were randomly divided into ten groups as shown in Figure 1. One group is a nonexposure group. Three groups were exposed to PM_10_ coal dust at doses of 6.25 (CD_6.25_), 12.5 (CD_12.5_), or 25 mg/m^3^ (CD_25_) an hour daily for 6 months. Six groups were exposed to coal dust with concomitant administration of *Eucheuma cottonii* at doses of 150 (EC_150_) or 300 mg/kg BW (EC_300_). The concentration of coal dust was determined according to occupational exposure in upper ground coal mining areas in South Kalimantan, Indonesia [21] and Turkey [22]. The doses of EC were based on previous study [16].

Coal dust exposure was performed as described in our previous study [20, 21]. The exposure chamber was designed and available in Laboratory of Pharmacology, Faculty of Medicine, Brawijaya University. The principal work of the chamber is to provide an ambient resuspended PM_10_ coal dust which can be inhaled by rats. Chamber size was 0.5 m^3^ and flowed by a 1.5–2 L/min airstream that resemble the environmental airstream. To prevent hypoxia and discomfort, we also provide oxygen supply in the chamber. Non-exposure group was exposed to filtered air in laboratory.

### 2.6. Tissue Sampling

At the end of the treatment, the animals were euthanized by anesthetizing with ether inhalation and exsanguinated by cardiac puncture. The lungs were collected, weighed, and washed with physiological saline. The right lung was histologically processed with hematoxylin-eosin staining and confocal microscopy (EGFR and MUC5AC). The left lung was homogenized to measure MDA by colorimetric and EGF, TGF-*α* by ELISA technique. All samples were labeled and stored at −80°C until analysis.

### 2.7. Analysis of Malondialdehyde

The lung MDA levels were measured by a modified method of Ohkawa et al. [23], based on the reaction of MDA with thiobarbituric acid (TBA) at 95°C in acid condition (pH 2-3), producing a pink pigment. Lungs were previously perfused free of blood with ice-cold PBS. Then, lungs were homogenized in KCl buffer (pH 7.6). The homogenate was mixed with 2.5 volumes of 10% (w/v) trichloroacetic acid to precipitate the protein. The precipitate was then centrifuged, and the supernatant was reacted with 0.67% TBA in a boiling water bath for 25 min. After cooling, the absorbance of the colored product was read at 532 nm using the spectrophotometer. The values obtained were compared with a series of MDA tetrabutylammonium salt (Sigma-Aldrich, St. Louis, MO, USA) standard solutions.

### 2.8. Analysis of EGFR Ligands

The serum TGF-*α* was measured using Rat TGF-*α* ELISA kits from NovaTeinBio. Inc. (Cambridge, MA, USA). The serum EGF ELISA kit was purchased from USCNK, Life Science. Inc. (Wuhan, Hubei, China). The analysis was done according to detail procedures in the kit.

### 2.9. Double-Labeling Immunofluorescence Staining of EGFR and MUC5AC

Double-labeling immunofluorescence staining of EGFR and MUC5AC was done according modified of previous study [24]. Paraffin-embedded lung sections (10 *μ*m thick) were immunostained according to the manufacturer's instructions (Santa Cruz Biotechnology, Dallas, TX, USA). Briefly, lung sections were deparaffinized in xylene and dehydrated through graded ethanol series. Nonspecific protein binding was blocked with 2% skim milk powder in PBS at RT for 20 min, followed by washing with PBS. Next, lung sections were incubated with rabbit anti-EGFR polyclonal (Santa Cruz Biotechnology) and mouse anti-MUC5AC monoclonal (DakoCytomation, Glostrup, Denmark) antibodies at specified dilutions for 1 h, followed by washing with PBS. The primary antibody bindings were then detected with goat anti-rabbit rhodamine (Santa Cruz Biotechnology) and goat anti-mouse FITC (Santa Cruz Biotechnology) antibodies at specified dilutions for 1 h in the dark, followed by washing with PBS. All PBS wash steps consisted of three washes of 5 min each. The expressions of EGFR and MUC5AC were analyzed by counting fluorescent intensity of cells (in arbitrary units; AU) in five random high-power (×400) microscope fields. The fluorescent images were recorded under a confocal laser scanning microscope (Olympus).

### 2.10. Statistical Analysis

Data are presented as mean ± SD, and the differences between groups were analyzed using one-way analysis of variance (ANOVA) with SPSS 15.0 statistical package for Windows. Only probability values of *P* < 0.05 were considered statistically significant and later subjected to Tukey's post hoc test.

## 3. Results

### 3.1. Radical Scavenging Activity

The EC at concentration 100 *μ*g/mL showed a weak free radical scavenging (20.11%) in the DPPH assay compared to catechin at this concentration (86.08%). This finding means that EC exhibited only weak antioxidant effect (Table 1).

### 3.2. Lung Histology

The exposure of several doses of coal dust to rat lungs affected the lung histology, as seen in Figure 1. CD_6.25_ induced lung parenchyma edematous. This edematous process decreased in CD_12.5_ and became necrotic in CD_25_. Chronic coal dust exposure increased the diameter of alveolus lumen. Besides, massive inflammatory cells were found in all coal dust exposure groups. CD_25_ induces vasodilation and hemorrhage. The administration EC_150_ and EC_300_ was able to decreased the diameter of alveolus lumen similar to control, but the inflammatory cells were still exist. In addition, this supplementation is also able to minimize hemorrhagic process.

### 3.3. Analysis of Malondialdehyde

**Figure 2:**
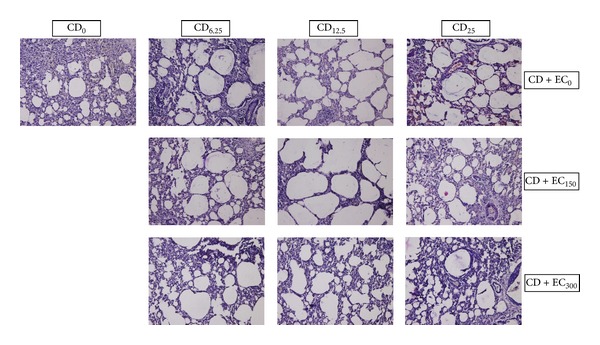
The morphology of lung in rats exposed to chronic coal dust and the effects of *E. Cottonii *supplementation (Hematoxyline Eosin staining, Magnification ×20). CD_6.25_ induced lung parenchym edematous. This edematous process decreased in CD_12.5_ and became necrosis in CD_25_. Chronic coal dust exposure increased the diameter of alveolus lumen. Besides, massive inflammatory cells were found in all coal dust exposure groups. CD_25_ induces vasodilation and hemorrhagic. The oral administration of EC_150_ and EC_300_ is able to decreased the diameter of alveolus lumen similar to control, but inflammatory cells were still exist. In addition, this supplementation also is able to minimizes the hemorrhagic process.

The exposure of several doses of coal dust to rat lungs affected the MDA levels, as shown in Figure 2. There were significantly (*P* < 0.05) increased MDA levels in groups exposed to coal dust at all doses compared to non-exposure group. The administration of EC_150_ significantly (*P* < 0.05) decreased the MDA levels in CD_6.25_ and CD_25_ groups compared to the respective coal dust-exposed nonsupplemented groups. The administration of EC_300_ significantly (*P* < 0.05) reduced the MDA levels in groups exposed to all doses of coal dust compared to the coal dust-exposed non-supplemented groups. 

### 3.4. Analysis of EGFR Ligand Levels

**Figure 3:**
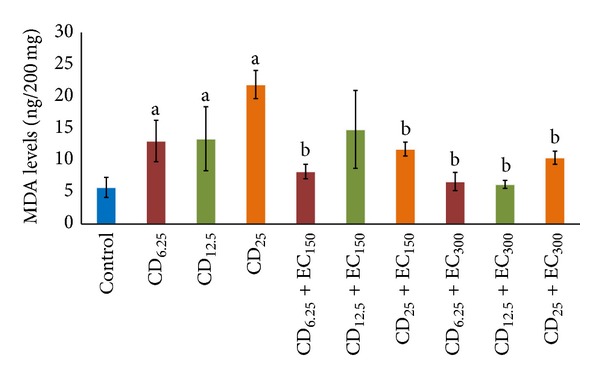
The levels of lung MDA. The lung MDA levels were increased in coal dust-exposed groups at all doses than that in non-exposure group but decreased in the *E. cottonii*-supplemented groups, except in CD_12.5_ + EC_150_ group. ^a^ *P* < 0.05 in comparison with non-exposure group, ^b^ *P* < 0.05 in comparison with its coal dust-exposed nonsupplemented group. Non-exposure group (control); group exposed to coal dust at dose of 6.25 mg/m^3^ (CD_6.25_), 12.5 mg/m^3^ (CD_12.5_), or 25 mg/m^3^ (CD_25_); group supplemented with the ethanolic extract of* E. cottonii* at dose of 150 (EC_150_) or 300 mg/kg BW (EC_300_).

The exposure of several doses of coal dust to rat lungs affected the EGF levels, as shown in Figure 3. There were significantly (*P* < 0.05) increased EGF levels in CD_12.5_ and CD_25_ groups compared to non-exposure group. Compared to the respective coal dust-exposed non-supplemented groups, the administration of EC_150_ and EC_300_ reduced the EGF levels in groups exposed to all doses of coal dust. However, the findings were not statistically significant.

**Figure 4:**
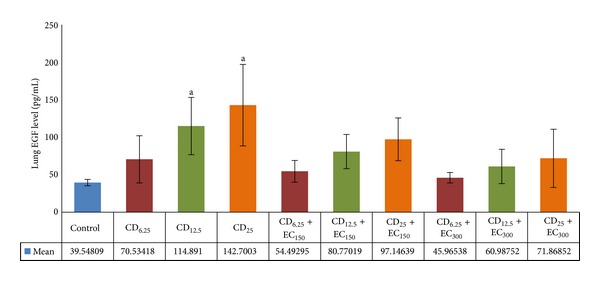
The levels of lung EGF. The lung EGF levels were increased in coal dust-exposed groups at doses of 12.5 and 25 mg/m^3^ than that in non-exposure group but decreased in the respective *E. cottonii*-supplemented groups. ^a^ *P* < 0.05 in comparison with non-exposure group, ^b^ *P* < 0.05 in comparison with its coal dust-exposed non-supplemented group. Non-exposure group (control); group exposed to coal dust at dose of 6.25 mg/m^3^ (CD_6.25_), 12.5 mg/m^3^ (CD_12.5_), or 25 mg/m^3^ (CD_25_); group supplemented with the ethanolic extract of* E. cottonii* at dose of 150 (EC_150_) or 300 mg/kg BW (EC_300_).

The exposure of several doses of coal dust to rat lungs affected the TGF-*α* levels, as shown in Figure 4. There was significantly (*P* < 0.05) increased TGF-*α* level in CD_25_ group compared to non-exposure group. Compared to its coal dust-exposed non-supplemented group, the administration of EC_150_ insignificantly decreased the TGF-*α* level in CD_6.25_ and CD_12.5_ groups, whereas EC_300_ insignificantly decreased the TGF-*α* level in groups exposed to all doses of coal dust.

### 3.5. Analysis of EGFR Expression

**Figure 5:**
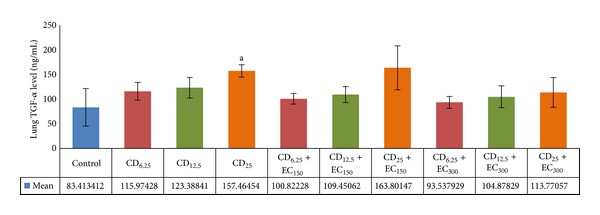
The levels of lung TGF-*α*. The lung TGF-*α* level was increased in coal dust-exposed group at dose of 25 mg/m^3^ than that in nonexposure group but decreased by supplementation of *E. cottonii* at dose of 300 mg/kg BW. ^a^ *P* < 0.05 in comparison with non-exposure group, ^b^ *P* < 0.05 in comparison with its coal dust-exposed non-supplemented group. Non-exposure group (control); group exposed to coal dust at dose of 6.25 mg/m^3^ (CD_6.25_), 12.5 mg/m^3^ (CD_12.5_), or 25 mg/m^3^ (CD_25_); group supplemented with the ethanolic extract of* E. cottonii* at dose of 150 (EC_150_) or 300 mg/kg BW (EC_300_).

The exposure of several doses of coal dust to rat lungs affected the EGFR expressions, as shown in Figure 5. The EGFR expressions were significantly (*P* < 0.05) increased in CD_12.5_ and CD_25_ groups compared to non-exposure group. Although not statistically significant, EC_150_ and EC_300_ (Figure 7) reduced the EGFR levels in groups exposed to all doses of coal dust compared to the respective coal dust-exposed non-supplemented groups.

### 3.6. Analysis of MUC5AC Expression

**Figure 6:**
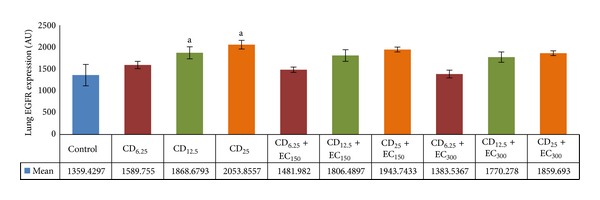
The expressions of lung EGFR. The lung EGFR expressions were increased in coal dust-exposed groups at doses of 12.5 and 25 mg/m^3^ than that in non-exposure group but decreased by supplementation of *E. cottonii* at dose of 300 mg/kg BW. ^a^ *P* < 0.05 in comparison with non-exposure group, ^b^ *P* < 0.05 in comparison with its coal dust-exposed non-supplemented group. Non-exposure group (control); group exposed to coal dust at dose of 6.25 mg/m^3^ (CD_6.25_), 12.5 mg/m^3^ (CD_12.5_), or 25 mg/m^3^ (CD_25_); group supplemented with the ethanolic extract of* E. cottonii* at dose of 150 (EC_150_) or 300 mg/kg BW (EC_300_).

**Figure 7:**
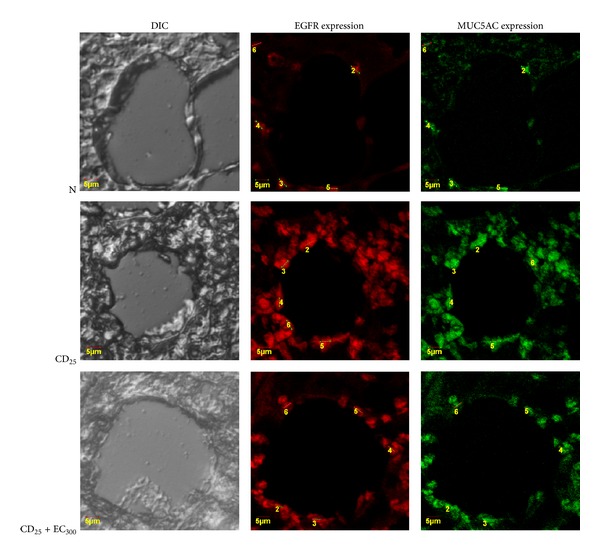
Representative immunofluorescence with anti-EGFR and anti-MUC5AC antibodies for determination of the lung EGFR and MUC5AC expressions in rats. These expressions were analyzed by counting fluorescent intensity of cells (in arbitrary units (AU)) in five random high-power (×400) microscope fields. The fluorescent images were recorded under a confocal laser scanning microscope. Cells were shown EGFR positive (*red fluorescent*) and MUC5AC positive (*green fluorescent*). Differential interference contrast (DIC); non-exposure group (N); group exposed to coal dust at dose of 25 mg/m^3^ (CD_25_); group supplemented with the ethanolic extract of* E. cottonii* at dose of 300 mg/kg BW (EC_300_).

The exposure of several doses of coal dust to rat lungs affected the MUC5AC expressions, as shown in Figure 6. The MUC5AC expression was significantly increased in CD_25_ group compared to non-exposure group, but EC_300_ is also able to reduce the MUC5AC expression in coal dust-exposed groups (Figure 7).

## 4. Discussion

In the present study, we observed a significant increase in MDA levels in rat lungs chronically exposed to coal dust. The MDA is a decomposition product of peroxidized polyunsaturated fatty acids that is widely preferred for detection of ROS reactivity toward lipid peroxidation [25, 26]. The severity of lipid damage is related to the concentration of oxidants in the tissue and hence to the efficiency of lipid repair mechanisms. The concentration of active metals and inhibitors also determines the severity of lipid damage. Coal dust redox reactivity is determined by its inorganic components and the size of particulate matter [21]. This study revealed that the administration of EC significantly (*P* < 0.05) decreased MDA levels in coal dust-exposed groups. This finding indicates that EC acts as an antioxidant *in vivo* to diminish the oxidative stress in lungs exposed to coal dust. The antioxidant mechanisms of EC, at least a part, are due to scavenging free radical activity.

Oxidative stress may regulate gene expression at both transcriptional and post-transcriptional levels [10]. Oxidative stress regulates MUC5AC mRNA expression via activation of EGFR [8, 9] and by an alternative mechanism, post-transcriptional regulation [10]. We have found that the levels of EGF and TGF-*α* as ligands for EGFR were significantly increased in coal dust-exposed group compared to nonexposure group (*P* < 0.05). In addition, the expressions of EGFR and MUC5AC were also significantly higher in coal dust-exposed group compared to non-exposure group (*P* < 0.05). This finding indicates that the ligand, receptor, and signaling for MUC5AC are upregulated in chronic coal dust exposure. Upregulation of these ligand involved the activity of metalloproteinase [27], mediated by inorganic component from coal dust. Compared to the respective coal dust-exposed non-supplemented groups, the administration of EC_150_ and EC_300_ reduced the EGF and TGF-*α* levels in groups exposed to all doses of coal dust. However, the findings were not statistically significant. Confocal micrograph showed that CD_25_ increased MUC5AC expression, but EC_300_ is able to diminish it. This finding indicated that EC_300_ is able to modulate the signaling for MUC5AC expression, at least a part, via decreasing the ligand production. The cysteine switch by active substances of EC is the one mechanism of ligand production inhibition [27]. Overall, the administration of *E. cottonii* is able to reverse the remodelling process in the lung exposed to chronic coal dust, especially the narrowing of alveolus lumen as early process to emphysema.

In conclusion, we found that chronic coal dust exposure increases oxidative stress and the signaling pathway induces mucin synthesis in rat lungs. The ethanolic extract of *E. cottonii* is able to decrease oxidative stress and signaling for mucin synthesis, at least a part, via reducing the ligand.

## Figures and Tables

**Table 1:** Radical scavenging activity of ethanolic extract of *E. cottonii*.

	Radical scavenging activity in %				
Concentration (*µ*g/mL)	6.25	12.50	25	50	100

Ethanolic extract of *E. cottonii *	0.59	8.04	14.70	16.28	20.11
Catechin	84.02	85.91	86.77	86.77	86.08
